# Automated vs manual cardiac MRI planning: a single-center prospective evaluation of reliability and scan times

**DOI:** 10.1007/s00330-025-11364-z

**Published:** 2025-01-22

**Authors:** Carl Glessgen, Lindsey A. Crowe, Jens Wetzl, Michaela Schmidt, Seung Su Yoon, Jean-Paul Vallée, Jean-François Deux

**Affiliations:** 1 Department of Radiology, Geneva University Hospitals, Geneva, Switzerland; 2 Siemens Healthineers AG, Forchheim, Germany

**Keywords:** Artificial intelligence, Cardiac imaging techniques, Magnetic resonance imaging, Software validation, Workflow

## Abstract

**Objectives:**

Evaluating the impact of an AI-based automated cardiac MRI (CMR) planning software on procedure errors and scan times compared to manual planning alone.

**Material and methods:**

Consecutive patients undergoing non-stress CMR were prospectively enrolled at a single center (August 2023–February 2024) and randomized into manual, or automated scan execution using prototype software. Patients with pacemakers, targeted indications, or inability to consent were excluded. All patients underwent the same CMR protocol with contrast, in breath-hold (BH) or free breathing (FB). Supervising radiologists recorded procedure errors (plane prescription, forgotten views, incorrect propagation of cardiac planes, and field-of-view mismanagement). Scan times and idle phase (non-acquisition portion) were computed from scanner logs. Most data were non-normally distributed and compared using non-parametric tests.

**Results:**

Eighty-two patients (mean age, 51.6 years ± 17.5; 56 men) were included. Forty-four patients underwent automated and 38 manual CMRs. The mean rate of procedure errors was significantly (*p* = 0.01) lower in the automated (0.45) than in the manual group (1.13). The rate of error-free examinations was higher (*p* = 0.03) in the automated (31/44; 70.5%) than in the manual group (17/38; 44.7%). Automated studies were shorter than manual studies in FB (30.3 vs 36.5 min, *p* < 0.001) but had similar durations in BH (42.0 vs 43.5 min, *p* = 0.42). The idle phase was lower in automated studies for FB and BH strategies (both *p* < 0.001).

**Conclusion:**

An AI-based automated software performed CMR at a clinical level with fewer planning errors and improved efficiency compared to manual planning.

**Key Points:**

***Question*** *What is the impact of an AI-based automated CMR planning software on procedure errors and scan times compared to manual planning alone*?

***Findings*** *Software-driven examinations were more reliable (71% error-free) than human-planned ones (45% error-free) and showed improved efficiency with reduced idle time*.

***Clinical relevance*** *CMR examinations require extensive technologist training, and continuous attention, and involve many planning steps. A fully automated software reliably acquired non-stress CMR potentially reducing mistake risk and increasing data homogeneity*.

## Introduction

Cardiac magnetic resonance imaging (CMR) is integral to modern cardiovascular diagnostics [1], yet its execution is demanding. Therefore, extensive MR technologist training is required and recommended by professional societies [2, 3]. Among other tasks, the technologist performing the procedure must find the reference cardiac planes, adjust all parameters for each sequence, and potentially interact with the patient during the same limited time. This complexity is reflected in the number of clicks required to perform an examination, an order of magnitude higher than non-CMR procedures [4]. The relative difficulty of these challenges can negatively impact the examination. First, they introduce a significant risk of planning errors, which prolong already extensive scan times at the cost of patient discomfort. Second, the repetitive setting up of sequence parameters focuses the technologist away from the quality assessment of previously acquired images, potentially limiting their diagnostic value. Finally, planning mistakes and inter-operator variability lead to heterogeneous datasets, compromising longitudinal comparisons of clinical cases and standardized research protocols. Thus, fully automated planning software could represent a pivotal shift in the field by completely automating the selection and monitoring of acquisition parameters, thereby reducing the likelihood of human error. Several research works have assessed automated cardiac plan prescription frameworks with promising results [5–13] without reaching full automation. Recently, preliminary conference works [14–16] have introduced single-click methods to fully automate CMR procedures, but a comprehensive evaluation of a clinically integrated end-to-end framework has yet to be published for cardiac imaging.

In this work, we hypothesized that automating the planning of a CMR protocol can reduce errors and decrease scan time. For this purpose, we designed a prospective study to evaluate the impact of a previously introduced [14, 15] AI-based automated CMR planning software on procedure errors and scan times compared to manual planning alone. Thus, we aim to assess whether automated acquisition can match or surpass the manual approach regarding reliability (error rate) and efficiency (scan time).

## Methods

### Study population

**Fig. 1:**
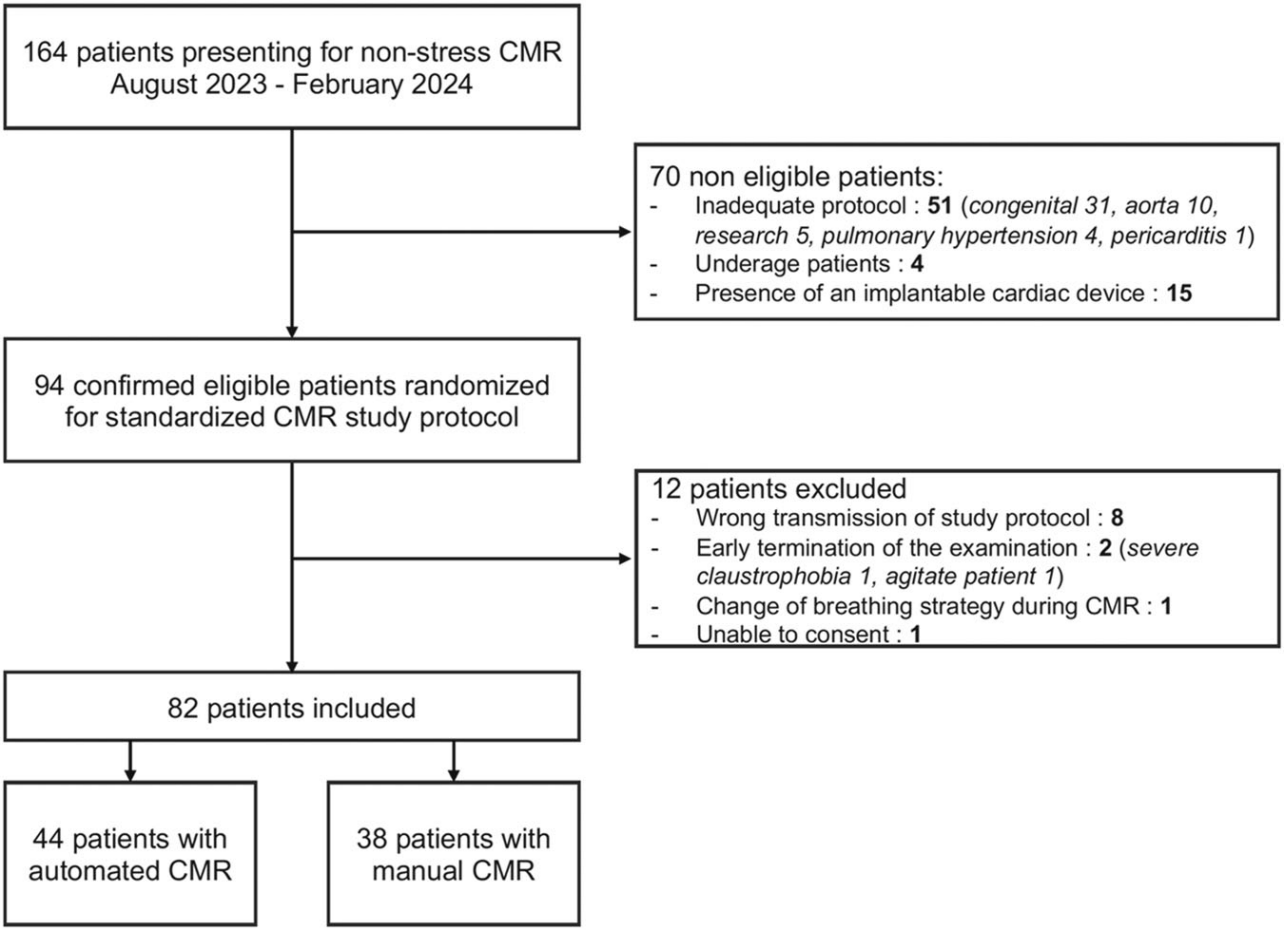
Study population flow-chart

The local ethical committee approved this prospective study, and all enrolled patients gave informed consent. Recruitment spanned from August 2023 to February 2024. During this period, consecutive patients scheduled for non-stress CMR on the scanner equipped with the software were considered potentially eligible. Patients with clinical indications requiring targeted sequences or planes not acquired in the evaluated protocol, patients under 18 years, and patients with an implantable cardiac device were excluded. On the examination day, inability or unwillingness to provide study consent, early CMR termination by the patient, change in breathing strategy during the examination (i.e., incorrect assessment of patient breath-hold (BH) capacity visible from breathing artifacts on first images), or wrong protocol initiated by the operating technologist led to patient exclusion. Included participants were randomized by alternating weekly between automated (software-driven acquisitions) and manual (human-driven acquisitions) study arms. The weekly alternation between automated and manual schemes allowed to streamline logistics and to reduce the cognitive load and error potential for technologists. The patient flow chart is available in Fig. 1.

### Software

A vendor-specific combination of a product (myExam Cardiac Assist) and research software (AI Cardiac Scan Companion, Siemens Healthineers AG) installed on the scanner was used to conduct the automated CMR acquisition. With this modular software, a pre-defined cardiac protocol can be run in an automated fashion. While the product software streamlines specific planning steps (e.g., automatic propagation of changes to following imaging sequences, auto-align cardiac views), this framework with additional AI-based functionality automatizes all steps required to conduct a CMR [14, 15]. Briefly, an Auto-Positioning technology identifies the center of the heart to move the table to the isocenter, as well as the positioning of the shim box, the image and diaphragm navigator, and the saturation bands. Second, it positions the standard localizers to perform Auto-Align without any need for manual interactions. Typical sequence parameters (i.e., field-of-view, number of acquired k-space lines, size of cine block, etc.) are adapted automatically to body size and cardiac rhythm by myExam Cardiac Assist. Finally, the inversion time (TI) for late enhancement imaging is identified automatically by an Auto-TI research framework [15] based on myocardium and blood pool intensities. At any point, the operator can intervene and fine-tune parameters, but only safety interruptions (checks for full contrast administration before proceeding) require human validation.

### CMR protocol

All examinations were performed on the same 1.5-T scanner (MAGNETOM Sola, Siemens Healthineers AG, software version XA51) and supervised by one of three radiologists (equivalent to SCMR level of 2, 3, and 3, respectively). Technologists were all trained in CMR but performed these examinations intermittently within a range of other MRI procedures. Clinical indication of the examination, patient compliance (optimal, acceptable, and low) and heart rate (< 70 bpm, 70–86 bpm, > 86 bpm), heart rhythm type (irregular in case of atrial fibrillation or extrasystoles, regular otherwise), and specific CMR experience of the technologist (low: < 2 years, mid-level: 2–5 years, and high: > 5 years) were recorded. The examination’s breathing strategy—either free breathing (FB) or BH—was chosen by the technologist based on each patient’s BH capacity. BH was performed in end-expiration to enable cardiac plane propagation between sequences acquired in BH or FB.

**Fig. 2:**
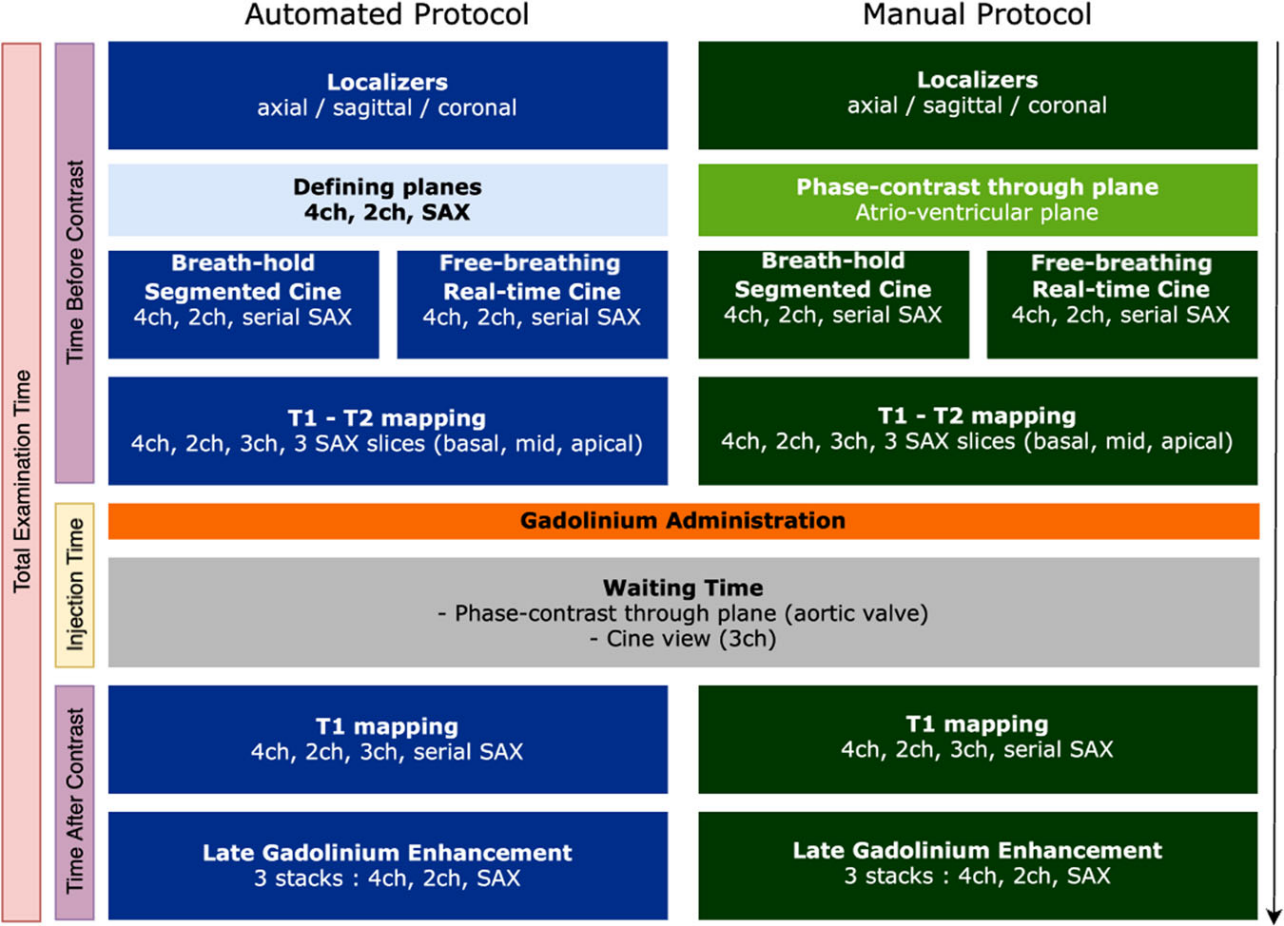
Overview of both protocols and views acquired in chronological order. On the left, the total examination time includes the time before contrast, injection time, and time after contrast. 4ch, 4-chamber view; 2ch, 2-chamber view; 3ch, 3-chamber view; SAX, short-axis stack

The same CMR protocol was performed in both automated and manual groups, with two exceptions. First, the automated protocol acquired additional localizers in the reference cardiac planes (long and short-axis views) after the initial localizers. Second, and in accordance with our local practice of optimizing cardiac plane identification, manual protocols included a phase-contrast (PC) sequence on the atrioventricular valve plane acquired after the initial localizers. All further sequences were identical between automated and manual protocols, overview of both protocols and the exact planes acquired for each sequence are represented in Fig. 2. Details of CMR sequences and the acquisition process are provided in the supplementary materials.

### Planning quality and scan times assessment

**Fig. 3:**
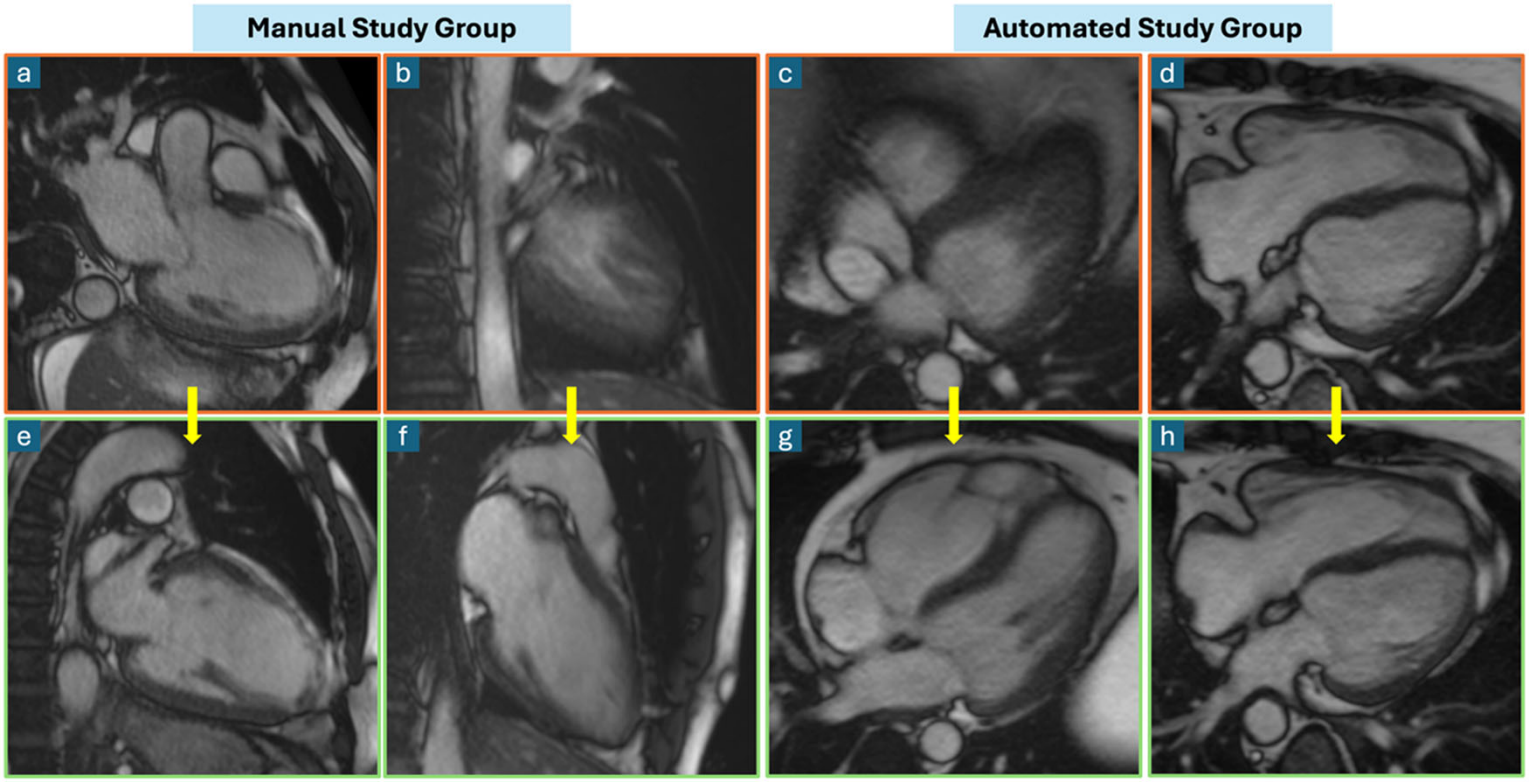
Showcase of plane prescription errors and their subsequent correction on balanced steady-state free precession cine images. Incorrect 2-chamber views (**a**, **b**) placement in the manual study group and their corrections (**e**, **f**). Incorrect 4-chamber views (**c**, **d**) placement in the automated study group and their corrections (**g**, **h**). Planning mistakes include a tilted axis into a 3-chamber instead of a 2-chamber view (**a**), a view outside the left ventricle (**b**, **c**), and a slightly misaligned view with poor visualization of the left atrium (**d**)

The supervising radiologists verified each acquired cardiac view in both automated and manual arms in real-time. As images were acquired, each of them was reviewed on a satellite console. In case of a procedure error, the sequence was manually adjusted and repeated. The number and type of procedure errors were recorded using the following classification: plane prescription error (requiring adjustment), forgotten view (only in the manual arm), incorrect propagation of a cardiac plane on a following sequence (wrong view), and field-of-view mismanagement (requiring repetition). Procedures without any errors and no need for repetitions were defined as error-free examinations. Planning quality by number of errors per procedure was compared between groups, both overall and across technologist experience levels. Examples of cardiac planes and adjustments made are presented in Fig. 3.

A blinded reading of all first cardiac planes produced (2-, 3-, and 4-chamber views, as well as short-axis stack) was performed separately (> 3 months) from the supervision by two independent readers (equivalent to SCMR levels 2 and 3). Each view was graded as follows: 1 (insufficient, needing correction), 2 (adequate with minor misalignments or obliquity), and 3 (impeccable). In addition, also at a distance (> 3 months) from the examinations, the inter-reader assessment of the software’s Auto-TI predictions was performed independently by one radiologist (SCMR level 2).

Timestamps from the examination were recorded directly from the scanner’s log and the following time blocks were computed: total examination time (from first to last image), time before contrast (from first to last pre-contrast image), injection time (from last pre-contrast to first post-contrast image), and time after contrast (from first post-contrast to last image). Acquisition time was computed from the cumulated sequence runtime. In opposition, the Idle phase was defined as the cumulated intervals between sequence acquisition (for examination planning, scanner adjustments, and inter-sequence breathing breaks) and computed for the following time blocks: total idle phase, idle phase before contrast, and idle phase after contrast. Finally, the cumulated Idle phase was computed at five successive time points (after localizers, cines, pre-contrast mapping, post-contrast mapping, and late gadolinium enhancement). Scan times were compared between groups, both overall and across technician experience levels.

### Statistical analysis

Data management and statistical analyses were conducted using Python (version 3.11, Python Software Foundation), with a significance level set at *p* < 0.05. The normality of the distribution of continuous variables and TI values was tested using the Shapiro–Wilk test. Age was the only variable showing a normal distribution (*p* = 0.07) and is therefore expressed as mean ± standard deviation; other non-normal variables are reported as median (25th–75th percentile), or averages. Continuous standard variables were compared by independent samples of the Student’s *t*-test and non-normal variables using the Mann–Whitney *U*-test. Categorical variables are summarized as counts and percentages. Comparisons of categorical data were performed using Pearson’s Chi-squared test with Yates’ continuity correction. A statistical comparison was deemed not applicable for categorical variables with fewer than five observations in any given category, and findings were annotated accordingly if applicable.

The inter-rater agreement for blinded reading of cardiac planes between the two independent readers was calculated using Cohen’s Kappa statistic. Both readers’ scores were subsequently averaged and compared between automated and manual groups using the Mann–Whitney *U*-test. The agreement between the software’s Auto-TI predictions and the TI predicted by the radiologist was assessed using Bland–Altman methodology, while statistical significance was evaluated using the Wilcoxon signed-rank test.

## Results

### Study population

One hundred sixty-four patients were considered for inclusion during the study period. After applying the exclusion criteria, 82 patients were finally included: 44 (54%) in the automated group and 38 (46%) in the manual one. The flowchart is represented in Fig. 1. No patients had congenital heart diseases with anatomical variants. Demographics, patient characteristics, technologist level, and breathing strategies were similar between groups, Table 1. The number of short-axis slices acquired was larger in automated procedures, with a median of 16 slices compared to 15 slices for manual procedures (*p* = 0.03).

**Table 1:** Demographical and clinical characteristics by study group

Parameters	Automated (*n* = 44)	Manual (*n* = 38)	*p*-value
Age (years)	50.30 ± 17.07	53.16 ± 18.22	0.52
BMI (kg/m^2^)	24.5 (21.8–27.5)	24.2 (22.0–28.0)	0.64
Sex			
Male	30 (68.2%)	26 (68.4%)	1.00
Female	14 (31.8%)	12 (31.6%)	
CMR indication			
Myocarditis	11 (25.0%)	11 (28.9%)	0.88
Infiltrative disease	9 (20.5%)	8 (21.1%)	1.00
Rhythm disorder	4 (9.1%)	4 (10.5%)	N/A
Cardiac viability	8 (18.2%)	9 (23.7%)	0.73
Structural evaluation	11 (27.3%)	6 (15.8%)	0.33
Patient compliance			
Optimal	35 (79.6%)	33 (86.8%)	0.56
Acceptable	6 (13.6%)	5 (13.2%)	1.00
Low	3 (6.8%)	0 (0.0%)	N/A
Heart rate (bpm)			
< 70	26 (59.1%)	23 (60.5%)	1.00
70–86	12 (27.3%)	12 (31.6%)	0.85
> 86	6 (13.6%)	3 (7.9%)	N/A
Heart rhythm			
Regular	37 (84.1%)	32 (84.2%)	1.00
Irregular	7 (15.9%)	6 (15.8%)	1.00
Technologist experience			
< 2 years	8 (18.2%)	6 (15.8%)	1.00
2–5 years	18 (40.9%)	11 (28.9%)	0.37
> 5 years	18 (40.9%)	21 (55.3%)	0.28
Breathing strategy			
BH	28 (63.6%)	21 (55.3%)	0.59
FB	16 (36.4%)	17 (44.7%)	
Number of short-axis slices	16.0 (15.0–17.0)	15.0 (14.0–16.0)	0.03

All data were collected by the supervising radiologist on standardized forms. Except for age, which is expressed as mean ± standard deviation, continuous values are expressed as median (IQR). The number of short-axis slices corresponds to the number of short-axis slices acquired for cine, post-contrast mapping, and late gadolinium enhancement

*BMI* body mass index, *bpm* beats per minute

### Assessment of examination quality

**Fig. 4:**
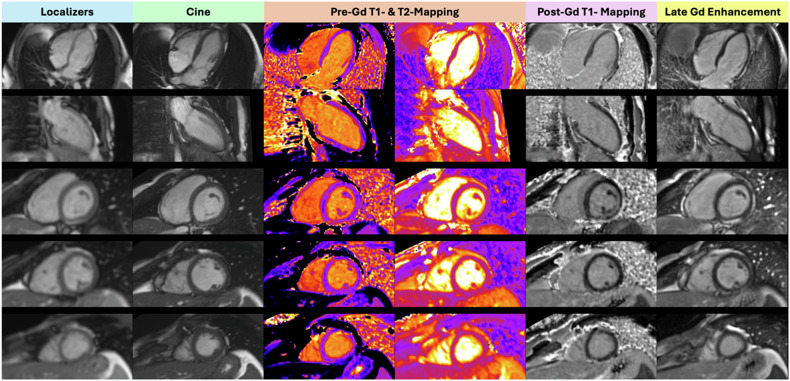
Selection of key images and blocks of a CMR examination acquired automatically by the software, with no recorded procedure error and a total examination time of 33 min. The patient was a 28-years-old male sent to rule out myocarditis

In both automated and manual groups, all examinations were complete and considered diagnostic (Fig. 4). In total, 43 errors were recorded in the manual and 20 in the automated groups. The number of procedure errors per examination was significantly (*p* = 0.01) higher in the manual group (1.13 errors per procedure) than in the automated group (0.45 errors per procedure). There was no significant difference between groups regarding plane prescription errors (*p* = 0.24). The rate of error-free examinations was significantly higher (*p* = 0.03) in the automated group (31/44; 70.5%) than in the manual group (17/38; 44.7%).

The number of procedure errors per examination remained non-significantly different between groups when stratified by technologist experience levels, except for mid-level technologists who showed more procedure errors per examination (1.36) in the manual group than in the automated group (0.44, *p* = 0.008). In the manual group, the rate of errors per procedure among high-level technologists (0.95) was lower than that of low- and mid-level technologists (1.33 and 1.36, respectively). In the automated group, there was a slight increase in errors per procedure with experience (from 0.25 to 0.56). All data are reported in Table 2.

**Table 2:** Procedure errors between automated and manual study groups

Parameter	Automated (*n* = 44)	Manual (*n* = 38)	*p*-value
Procedure errors *n* (errors per procedure)			
Total	20 (0.45)	43 (1.13)	0.01
Plane prescription errors	19 (0.43)	26 (0.68)	0.24
Forgotten views	0 (0)	9 (0.24)	0.007
Incorrect plane propagations	1 (0.02)	4 (0.10)	0.12
Field-of-view mismanagements	0 (0)	4 (0.10)	0.06
Procedure errors by technologist experience *n* (errors per procedure)			
Low	2 (0.25)	8 (1.33)	0.37
Mid-level	8 (0.44)	15 (1.36)	0.008
High	10 (0.56)	20 (0.95)	0.33
Error-free examinations *n* (%)	31 (70.5)	17 (44.7)	0.03

Values are expressed as count (mean error rate per examination) or count (% of all examinations)

There was strong agreement (κ = 0.68) between both readers for the blinded grading of cardiac planes. No significant difference in score was found between both groups, with a mean score of 2.81 for manual and 2.87 for automated examinations (*p* = 0.07). Inter-reader agreement between reference (human reading) and predicted (Auto-TI) TI values for myocardial nulling was excellent, showing no significant difference between human and software reading (*p* = 0.17). The Bland–Altman chart is available in the Supplementary Material.

### Evaluation of scan times

The Acquisition Time did not differ significantly between groups, but scan times in the automated workflow tended to be slightly longer for all patients (20.5 min vs 19.5 min) and BH examinations (22.1 min vs 19.9 min). The total examination time, Injection Time, and Time After Contrast showed no significant differences between automated and manual procedures. However, the Time Before Contrast was significantly lower in the automated group than in the manual group (16.6 min vs 19.0 min, corresponding to a 13%-time reduction, *p* = 0.04). In the subgroup of FB examinations, total examination time and time before contrast were significantly shorter in the automated group than in the manual group, with a relative 17%- and 43%-time reduction, respectively (both with *p* < 0.001). No significant difference was detected between automated and manual groups in BH examinations for any procedure duration. The total idle phase and the idle phase before contrast were significantly lower in the automated group compared to the manual group (both with *p* < 0.001). The idle phase after contrast showed no difference between groups (*p* = 0.54). Similarly, the differences in total idle phase and idle phase before contrast between groups were consistently observed across both FB and BH subgroups. Scan times are reported in Table 3.

**Table 3:** Overview of scan times across study groups

Parameter	Group	Automated (*n* = 44)	Manual (*n* = 38)	Δ	*p*-value
Total examination time (min)	All patients	38.5 (30.8–44.4)	41.2 (37.8–44.2)	−6%	0.13
	BH	42.0 (38.6–47.6)	43.5 (41.0–47.4)	−3%	0.42
	FB	30.3 (27.9–30.9)	36.5 (32.6–41.2)	−17%	< 0.001
Acquisition time (min)	All patients	20.5 (18.8–23.3)	19.5 (17.8–22.0)	+5%	0.17
	BH	22.1 (19.9–23.9)	19.9 (18.4–22.6)	+10%	0.07
	FB	18.9 (16.1–20.0)	18.9 (17.4–20.8)	–	0.67
Time before contrast (min)	All patients	16.6 (9.6–21.0)	19.0 (16.3–21.4)	−13%	0.04
	BH	19.3 (17.2–22.8)	20.8 (19.0–23.3)	−7%	0.21
	FB	9.0 (8.4–9.7)	15.9 (14.5–18.9)	−43%	< 0.001
Injection time (min)	All patients	5.1 (4.7–6.1)	5.0 (4.7–6.1)	+0.2%	0.85
	BH	4.8 (4.6–6.0)	5.0 (4.7–6.0)	−0.4%	0.64
	FB	5.6 (5.0–6.2)	5.1 (4.9–6.1)	+10%	0.59
Time after contrast (min)	All patients	16.5 (14.8–18.1)	15.8 (14.4–8.0)	+4%	0.37
	BH	17.3 (16.1–18.9)	16.9 (15.7–18.6)	+2%	0.72
	FB	15.2 (13.6–16.8)	14.4 (12.5–15.7)	+6%	0.36
Total idle phase (min)	All patients	12.6 (6.0–15.4)	16.2 (12.8–18.2)	−22%	< 0.001
	BH	14.8 (12.8–16.8)	17.9 (16.5–19.8)	−17%	< 0.001
	FB	5.7 (5.0–6.2)	12.6 (9.0–15.2)	−55%	< 0.001
Idle phase before contrast (min)	All patients	9.8 (4.7–12.6)	13.1 (11.1–15.0)	−25%	< 0.001
	BH	11.7 (10.1–13.6)	14.4 (13.2–16.8)	−19%	< 0.001
	FB	3.8 (3.5–4.9)	10.6 (8.0–12.9)	−64%	< 0.001
Idle phase after contrast (min)	All patients	2.5 (1.8–3.4)	2.3 (1.3–3.6)	+9%	0.54
	BH	3.0 (2.2–4.1)	3.5 (2.2–3.6)	−14%	0.95
	FB	1.6 (1.1–2.2)	1.3 (0.6–2.1)	+23%	0.36

Procedure durations and idle phase components of each major time parameter as compared between the two study groups. All values are expressed as median (IQR)

Δ = Difference (%) between automated and manual groups

Analysis of idle phase stratified by technologist experience levels showed a significantly shorter idle phase before contrast across all levels, but statistical power was not achieved for the total idle phase except for mid-level technologists (*p* = 0.03), Table 4. Of note, the distribution of breathing strategies stratified by technologist experience and compared between study groups did not show any significant difference (Table 5 in the Supplementary Materials).

**Table 4:** Scan times stratified by technologist experience levels

Parameter	Technologist experience	Automated (*n* = 44)	Manual (*n* = 38)	Δ	*p*-value
Total examination time (min)	Low	40.7 (35.7–46.0)	44.9 (41.3–46.3)	−9%	0.66
	Mid-level	40.0 (31.0–43.0)	42.8 (39.1–49.6)	−7%	0.07
	High	36.0 (30.7–45.1)	40.6 (36.5–42.7)	−11%	0.66
Total idle phase (min)	Low	13.3 (10.3–15.3)	17.5 (16.3–20.9)	−24%	0.053
	Mid-level	13.1 (6.7–15.1)	16.3 (14.3–20.2)	−20%	0.03
	High	11.1 (5.9–16.3)	15.2 (12.6–16.8)	−28%	0.059
Idle phase before contrast (min)	Low	11.2 (8.3–12.7)	15.3 (12.7–18.2)	−27%	0.02
	Mid-level	9.9 (4.9–12.4)	14.5 (10.1–16.6)	−32%	0.01
	High	8.9 (4.3–13.0)	13.0 (10.6–13.9)	−32%	0.04
Idle phase after contrast (min)	Low	2.3 (1.4–3.0)	2.5 (1.9–3.3)	−8%	0.86
	Mid-level	2.7 (1.7–3.5)	2.4 (1.0–3.8)	+13%	0.79
	High	2.4 (2.0–3.5)	2.2 (1.3–3.6)	+9%	0.34

Procedure durations and idle phase components were stratified by technologist experience levels and compared between the two study groups. All values are expressed as median (IQR)

Δ = Difference (%) between automated and manual groups

**Fig. 5:**
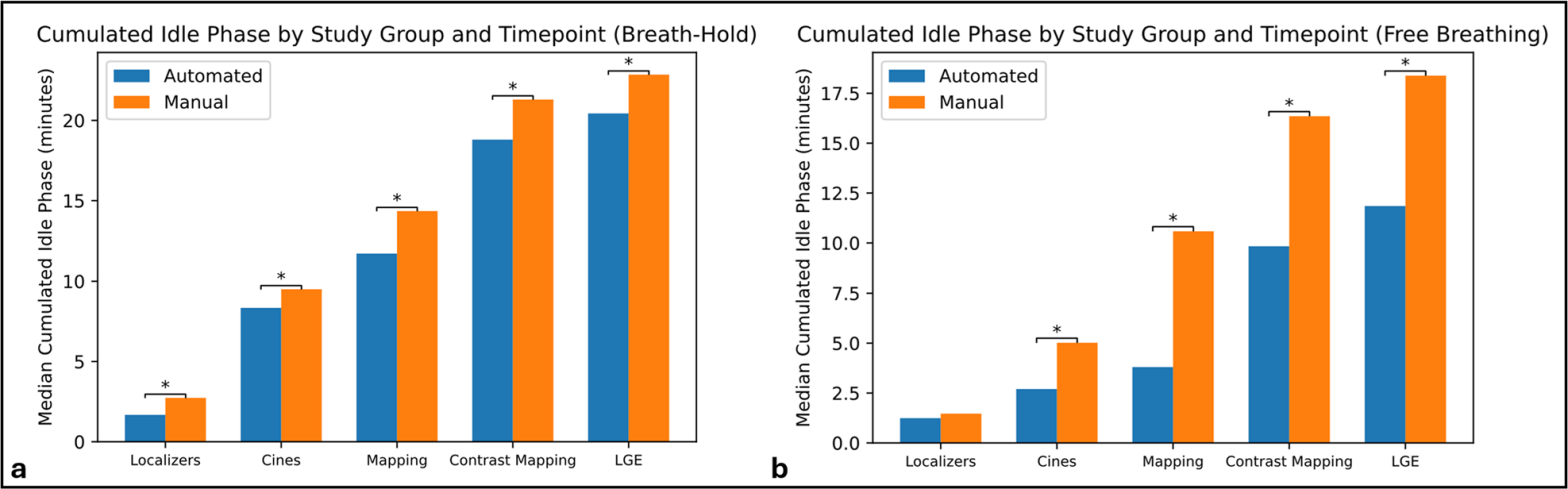
Cumulated idle phase throughout CMR examinations in breath-hold (**a**) and free breathing (**b**) at five different timepoints: after the localizers and plane-defining sequences, after the cine sequences, after mapping (T1 and T2), after contrast administration and post-contrast mapping (T1), and after late gadolinium enhancement (LGE). Asterisks signify significant differences in median values between protocols (*p* < 0.05)

Analysis at individual time points showed that the cumulated idle phase measured at five successive moments of the MR examination was significantly shorter in the automated group than in the manual group for all time points and both respiratory strategies, with one exception (the idle phase after localizers in FB), Fig. 5 (Table 6 in the Supplementary Materials).

## Discussion

In this prospective study, we evaluated an automated CMR planning software consisting of product and research functionality, aimed at alleviating the operator’s most repetitive and monopolizing tasks. We compared fully automatic and human-driven studies over planning quality and scan times outcomes. On a qualitative level, our results showed that the automatic procedures had fewer errors (∼71% of error-free procedures vs ∼45% without). In addition, it significantly reduced examination times by ∼6 min (17%) in FB examinations and increased efficiency by reducing the idle portion of examinations in both breathing strategies.

Artificial intelligence (AI) is transforming cardiac imaging with numerous applications in image reconstruction, analysis, and reporting [17]. However, in comparison to works addressing specific tasks related to CMR acquisition or post-processing [18–21], literature on fully automated CMR acquisition is relatively scarce. Efforts have been made to automate some parts of the procedure through different frameworks of autonomous cardiac plane prescription [5–13], but apart from preliminary studies [14–16] work has yet to report on software allowing complete examination automation as part of a routine clinical activity. To our knowledge, this is the first complete work validating a single-click CMR acquisition framework in a clinical environment.

We report that the software-driven procedures showed fewer errors than human-driven ones, virtually all due to plane prescription errors. In contrast, human errors were more frequent and heterogeneous due to plane errors and oversights in propagating planes, copying sequence parameters, and adapting field-of-views. The predictable error pattern of the software is advantageous as it reduces the cognitive load of quality checks for the operator, but also for physicians whose supervision might be spread on multiple scanners. The software performed on par with human operators regarding plane prescription and TI selection, two tasks that had been managed independently with previous frameworks but required a combination to fully drive a CMR acquisition. Of note, in the manual group, high-level technologists exhibited fewer errors per procedure than their less experienced counterparts, likely reflecting their advanced skill and familiarity with the process. Within the automated group, as technologists only provided supervision, variations in errors across experience are granular and not linked with technologist experience.

We report reductions in total examination time (up to 17%) and idle phase (up to 55%) in the automated workflow, despite the limitation that more short-axis slices were acquired compared to the manual one. Indeed, the software kept the number of short axis views between cine, mapping, and late enhancement constant, while technologists often reduced the number for mapping and late enhancement. Surprisingly, a significant reduction in total examination time was only observed in the FB subgroup. We hypothesize that in BH conditions, longer acquisition times and breathing breaks additionally masked the software’s higher efficiency and prevented significant time savings. The software’s potential efficiency is evident from within-group comparisons. Automated FB procedures showed a 72% lower Idle Phase than BH procedures (5.7 min vs 14.8 min), almost not relying on breathing breaks for planning. In contrast, human-driven procedures only showed a 30% drop in idle phase between FB and BH (12.6 min vs 17.9 min), suggesting that technologists, relying on external delays for planning, could not reduce idle phase as efficiently without them. Our results are consistent with a preliminary assessment of the technology evaluated in this study [14], reporting a 15%-time reduction. Meanwhile, Kwong and al. reported a 30% reduction in scan time using another AI technology, from 54 min to 38 min, although their exact protocols and respiratory strategies were not specified [16]. The more efficient 41-min overall average in our manual group might explain the disparity with their data, while our 38-min overall average in the automated group remains similar.

Automating MRI acquisitions is a big leap forward and our findings have several implications regarding diagnostics, professional training, and economics. For smaller non-academic hospitals or radiology departments wishing to develop cardiac imaging, having a plug-and-play framework is the first step to providing enhanced diagnostics. In our study, the software was able to cover 50% of all non-stress CMR indications. Second, it may represent an opportunity to teach technologists hands-on, particularly less experienced ones. Software taking care of repetitive tasks may allow them to better focus on the patient, the image quality, exchanges with the radiologist, or performing image post-processing. Finally, this study suggests a methodology to evaluate an MRI companion software, which might be helpful to other teams wishing to validate similar tools in different environments.

Our study had some limitations. The main one was that radiologists could not realistically be blinded to the study arm as automatic acquisitions were performed with almost no visible human interaction. To mitigate this bias, three different supervisors participated in the study, and a blinded analysis of cardiac planes was performed. Second, the number of included participants was relatively small which limits the generalization of our findings, particularly when addressing the relationship between technologist experience and study outcomes. Finally, we were not able to demonstrate a significant time reduction using the automated software in the BH subgroup (*p* = 0.42), most likely due to a small effect size and overlapping scan durations between automated and manual examinations. While a much larger sample size might have led to statistical significance, the effect size would remain too low to impact our findings, as the observed efficiency benefits and planning reliability with automated CMR are already clear.

In conclusion, automated CMR planning software could automatically carry out CMR procedures with a lower error rate than human planning and increased efficiency. Secondary benefits on patient workflow, technologist comfort, and image quality can be expected with this type of software and could be the subject of further studies. Also, additional consideration should be given to identifying which protocols, patients, and clinical situations would most benefit from the increased efficiency in FB conditions.

## Supplementary information

ELECTRONIC SUPPLEMENTARY MATERIAL

## Abbreviations

AI: Artificial intelligence
BH: Breath-hold
CMR: Cardiac MRI
FB: Free breathing
TI: Inversion time
