# Supplementary material for: Automated vs manual cardiac MRI planning: a single-center prospective evaluation of reliability and scan times
Source: Eur Radiol. 2025 Jan 22;35(7):3927–36. doi: 10.1007/s00330-025-11364-z (PMC12166016; doi:10.1007/s00330-025-11364-z)
Supplement: Supplementary file 1 — ELECTRONIC SUPPLEMENTARY MATERIAL [file 330_2025_11364_MOESM1_ESM.pdf]

Automated vs. manual cardiac MRI planning: a single-center prospective evaluation of reliability and scan times

ELECTRONIC SUPPLEMENTARY MATERIAL

CMR sequences and acquisition details

- True FISP localizers (typical parameters: TR 272 ms, TE 1.12 ms, flip angle 80°, ST 8 mm, FoV 399*399) were acquired in all three directions.
- After localizers, a phase contrast sequence was acquired through the atrio-ventricular valve plane in the manual group (through-plane, VENC 150 cm/sec). In the automated group, a second set of plane-defining localizers was acquired in 4- and 2-chamber as well as short axis views (typical parameters: TR 224 ms, TE 1.05 ms, flip angle 80°, ST 8 mm, FoV 499*499).
- Cines were used to analyze cardiac function and were acquired in 4- and 2-chamber views, as well as one serial, no-gap short-axis stack. In breath-hold, segmented balanced SSFP (steady state free precession) cines were acquired (typical parameters: TR 38 ms, TE 1.16 ms, flip angle 60°, ST 8 mm, FoV 360*330). In free-breathing, real-time compressed sensing (17) balanced SSFP cines were acquired (typical parameters: TR 34 ms, TE 1.05 ms, flip angle 59°, ST 8 mm, FoV 500*298).
- T1 and T2 mapping were both performed in 4-, 3- and 2-chamber views, and in three short axis slices, using a shortened MOLLI scheme and FLASH readouts for T1 and T2 mapping, respectively(18,19)). Inversion times for T1 mapping ranged from 110 to 5875 ms.
- Contrast material was administered intravenously (Dotarem, Guerbet, Villepinte, France: 0.02 mmol/Kg) at a low injection rate of 1-3 ml/sec depending on the situation.
- After contrast administration, a 4 four-minute waiting time was respected before starting post-contrast imaging. During this waiting time, phase contrast sequences were acquired through the aortic valve (through-plane, VENC 250 cm/sec) and in 3-chamber orientation (in-plane, VENC 250 cm/sec).

- Four minutes after contrast administration, post-contrast T1 mapping (4-, 3- and 2-chamber views in breath-hold, serial short-axis stack in free breathing) was acquired. Inversion times for post-contrast T1 mapping ranged from 100 to 2060 ms.
- A single short axis Look-Locker TI-scout was acquired before the late enhancement sequences at mid-ventricular level (typical parameters: TR 49 ms, TE 1.67 ms, flip angle 35°, ST 8 mm, FoV 126*208). Inversion times ranged from 125 to 575 ms.
- Free breathing late enhancement imaging phase-sensitive inversion recovery (PSIR) sequences(20) were acquired in three stacks: 4- and 2-chamber, short axis (typical parameters: TR 724 ms, TE 1.18 ms, flip angle 55°, ST 8 mm, FoV 360*284).
- In total, three stacks were acquired serially in short-axis orientation: cine stack, post-contrast T1 mapping stack, and PSIR stack. Due to identical slice parameters (8 mm thickness, no distance factor), the same number of slices was acquired for all three stacks in the automated group and was recorded for each patient. In the manual group, the number of slices for each block was recorded separately and averaged.

Inter-reader assessment of myocardial nulling TI identification

Bland-Altman chart comparing inversion times as identified by an independent reader and by the Auto TI framework of the AI Cardiac Scan Companion software being evaluated. Agreement between human reading and software reading was not significantly different (p = 0.17). Forty-two from 44 predicted times were within the limits of agreements, and 34/44 (77%) were strictly identical to the predicted time.

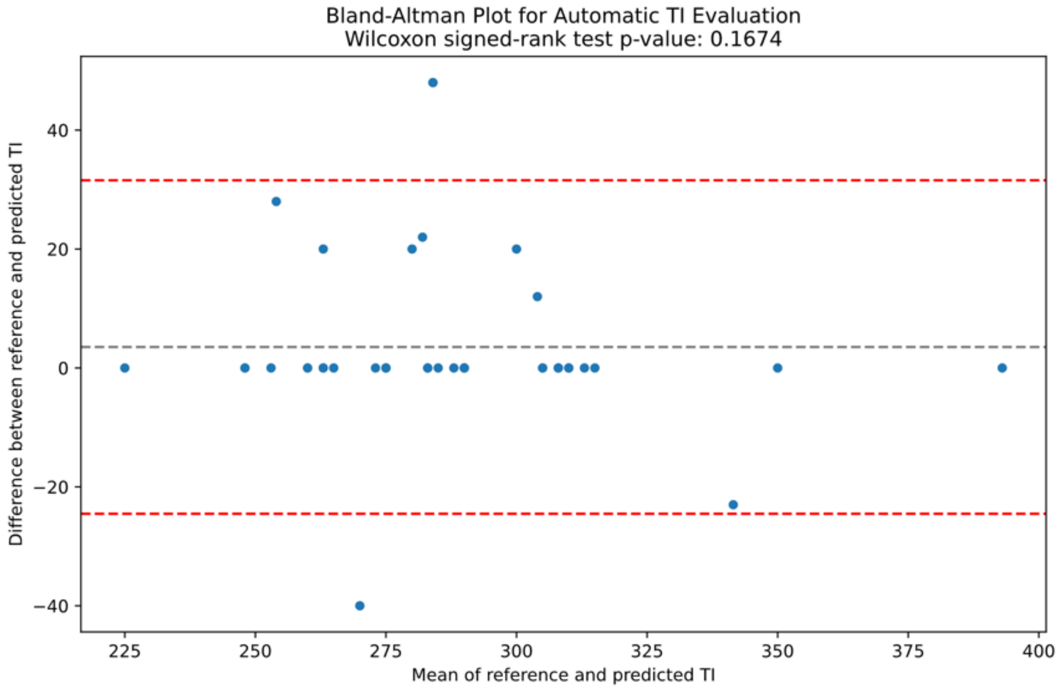
Bland-Altman Plot for Automatic TI Evaluation
Wilcoxon signed-rank test p-value: 0.1674
Difference between reference and predicted TI
Mean of reference and predicted TI
40
20
0
−20
−40
225
250
275
300
325
350
375
400

Distribution of breath-hold examinations across technician experience levels

**Technologist Experience**	**Proportion of breath-hold examinations (Automated)** Count from total (%)	**Proportion of breath-hold examinations (Manual)** Count from total (%)	p-value
Low	6 / 8 (75%)	4/6 (67%)	1.00
Mid-Level	12/18 (67%)	5/11 (52%)	0.46
High	10/18 (56%)	12/21 (57%)	1.00

Table 5. Proportion of breath-hold examinations compared between groups and stratified by technician level. No significant difference in breathing strategy can be observed across technician experience levels. Proportions are expressed as count from total cases in the group at this experience level.

Detailed analysis of cumulated Idle Phase at specific timepoints

Idle Phase	Breathing Strategy	**Automated (n = 44)**	**Manual (n = 38)**	p-value
After Localizers (min)	All patients	1.6 (1.3 - 1.8)	2.2 (1.5 - 3.1)	< 0.001
	BH	1.7 (1.6 - 1.9)	2.7 (2.0 - 3.5)	< 0.001
	FB	1.2 (1.1 - 1.3)	1.5 (1.2 - 2.2)	0.06
After Cines (min)	All patients	6.6 (3.4 - 9.3)	8.3 (5.4 - 10.1)	0.03
	BH	8.3 (6.8 - 9.8)	9.5 (8.4 - 11.4)	0.02
	FB	2.7 (2.3 - 3.5)	5.0 (4.4 - 5.9)	< 0.001
After Mapping (min)	All patients	10.0 (4.7 - 12.8)	13.0 (11.0 - 15.0)	< 0.001
	BH	11.7 (10.4 - 13.6)	14.3 (13.2 - 16.8)	< 0.001
	FB	3.8 (3.5 - 4.9)	10.6 (8.0 - 12.3)	< 0.001
After Post-contrast Mapping (min)	All patients	11.2 (4.8 - 13.9)	14.3 (11.5 - 16.3)	< 0.001
	BH	12.7 (11.6 - 14.6)	16.2 (14.6 - 18.6)	< 0.001
	FB	3.8 (3.5 - 5.0)	10.9 (8.1 - 12.3)	< 0.001
After Late Gadolinium Enhancement (min)	All patients	12.6 (6.0 – 15.4)	16.2 (12.8 – 18.2)	< 0.001
	BH	14.8 (12.8 – 16.8)	17.9 (16.5 – 19.8)	< 0.001
	FB	5.7 (5.0 – 6.2)	12.6 (9.0 – 15.2)	< 0.001

Table 6. Cumulated Idle Phase (minutes) after specific cardiac MRI procedure imaging blocks. All values are expressed as median (IQR).
